# Case report: Incomplete laceration of the right middle lobar bronchus due to blunt chest trauma: An unusual pattern and attentions

**DOI:** 10.3389/fsurg.2022.1011674

**Published:** 2022-09-05

**Authors:** Yuqi Song, Jianzun Ma, Jing Wang, Linan Fang

**Affiliations:** ^1^ Department of Thoracic Surgery, The First Hospital of Jilin University, Changchun, China; ^2^ Department of Radiology, The First Hospital of Jilin University, Changchun, China; ^3^ State Key Laboratory of Supramolecular Structure and Materials, College of Chemistry, Jilin University, Changchun, China

**Keywords:** blunt chest trauma, tracheobronchial laceration, hemopneumothorax, bronchoscopy, surgery

## Abstract

Tracheobronchial ruptures caused by blunt chest trauma are rarely encountered but may be life-threatening. It is even rarer when the rupture is in the right middle lobe bronchus. Here we present a case of incomplete laceration of the right middle lobe bronchus after blunt trauma, which could easily be overlooked because of the absence of obvious symptoms. A 58-year-old man suffered multiple traumas after being attacked by cattle, closed chest drainage was promptly performed in the local hospital for bilateral hemopneumothorax. Three days later, the patient was transferred to our center for urgent exploratory thoracic surgery due to persistent hemothorax. We did not diagnose bronchial injury even after a bedside emergency bronchoscopy due to the adherence of bloody secretions and sputum crusts. It was not until a repeat chest CT 4 days after the initial surgery that we suspected an incomplete right middle lobe bronchial laceration, which was confirmed by postoperative bronchoscopy. The patient eventually underwent right middle lobe lung resection for a deep and wide bronchial laceration and recovered well. Clinicians should be fully aware of the possibility of this condition after blunt chest trauma and make good use of CT and bronchoscopy to help with diagnosis and treatment.

## Introduction

Tracheobronchial tear due to blunt trauma is rare but could be life-threatening. We present a case of an incomplete laceration of the right middle lobe bronchus in a cattle-caused blunt trauma. Without typical clinical features, the diagnosis may be missed, which can be a challenging task for the emergency physician unless the physician suspects the possibility. Finally, the patient was successfully treated with a right middle lobectomy and subsequent intensive care.

## Case presentation

A previously healthy 58-year-old man was attacked by cattle while doing farm work and was immediately taken to the emergency room of a local hospital with chest pain, dyspnea, and lower extremity motor and sensory dysfunction. The computed tomography (CT) scan showed multiple rib fractures, bilateral hemothorax and pneumothorax, multiple thoracic vertebral fractures (T6,7,9), right clavicle and scapula fracture, and a subepithelial liver injury and renal injury. Bilateral closed chest drains were performed promptly, and hemorrhagic fluid was drained out. The patient was transferred to our hospital 3 days after the primary injury due to persistent drainage of hemorrhagic fluid and an air leak in the chest drain. His baseline vital signs on admission were T: 36.5°C, P: 105 beats/min, R: 22 breaths/min, and BP: 88/55 mmHg. Due to partial atrophy of the patient's right lung, CT did not show any significant rupture or laceration of the tracheobronchial tree. A bedside emergent bronchoscopy was performed; however, no evidence of bronchial rupture was found due to the presence of bloody secretions and sputum crusts. The patient's right chest drainage tube continued to drain hemorrhagic fluid and the hemoglobin dropped to 85 g/L. The patient was considered to have progressive hemopneumothorax and a right exploratory thoracotomy was performed urgently. Through the right fourth intercostal incision, a blood clot was found to have accumulated in the right thoracic cavity and a fractured rib had punctured the right lung. Blood clot removal, lung repair, open reduction, internal fixation of the rib fracture, and closed drainage of the chest cavity were then performed. Intraoperative exploration revealed no bronchial rupture and no evidence of air leak in the right lung was found.

**Figure 1:**
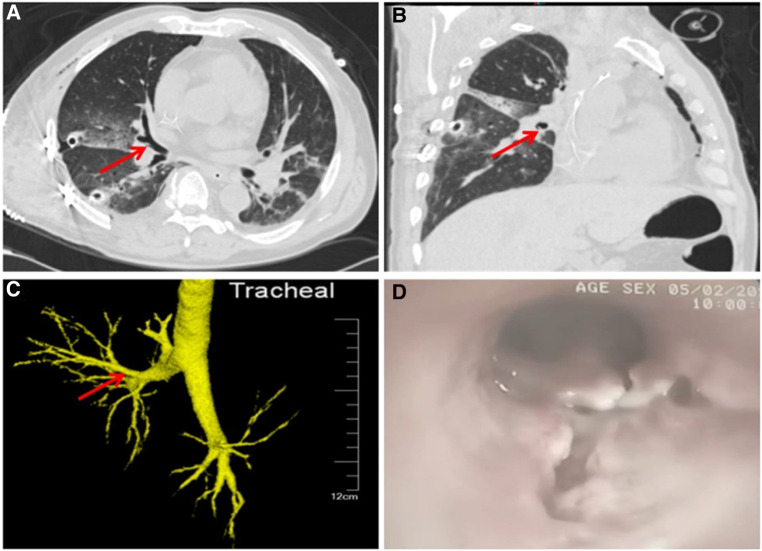
Axial (**A**) and sagittal (**B**) view of CT shows the laceration of the bronchial wall in the middle lobe of right lung. The red arrow indicates the location of the laceration. CT 3D reconstruction (**C**) shows that the bronchi are continuous and intact, while the laceration is clearly demonstrated in bronchoscopy (**D**).

**Figure 2:**
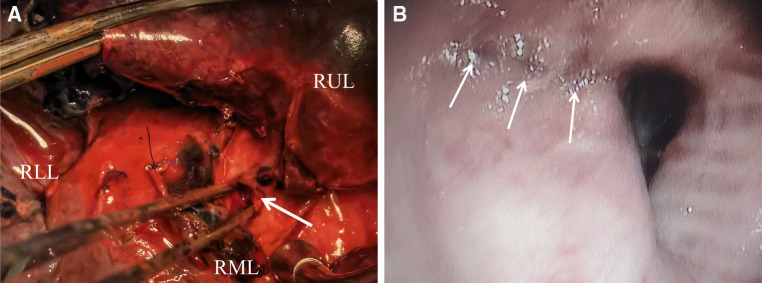
(**A**) Open surgical field. The arrow indicates the opening of the bronchial dissection seen during surgery. (**B**) Postoperative bronchoscopy confirmed good closure of the bronchial stump. RUL, right upper lobe; RML, right middle lobe; RLL, right lower lobe.

Postoperatively, no air leakage was found from the right chest tube drainage and the bedside chest roentgenogram showed that both lungs were well recruited. When the CT scan was repeated 4 days post-surgery, interruption of the right middle lobe endobronchial wall was found in the axial and sagittal view (Figures 1A,B) but chest CT with three-dimensional reconstruction shows no obvious rupture or laceration of the tracheobronchial tree (Figure 1C). Incomplete laceration of the right middle lobe bronchus was suspected, which was confirmed by a bedside bronchoscopy after removing the secretion and sputum in the patient's airway (Figure 1D). Due to the long interval between the patient's injuries, the severe bronchial laceration, the significant local inflammatory response, and the potential for long-term complications with conservative treatment, we decided to perform a second operation to resect the impaired middle lobe of the right lung. During the procedure, we found that the middle lobe bronchus was incompletely disrupted, as the small laceration became visible only after the removal of the tissue and cystic membrane surrounding the middle lobe bronchus (Figure 2A). The surgery was timely and successful, and postoperative bronchoscopy confirmed good closure of the bronchial stump (Figure 2B). The patient recovered gradually and he was then transferred to the spinal surgery department for further treatment.

## Discussion

Tracheobronchial rupture due to blunt thoracic trauma is rare but is associated with high mortality. In a review of 1,178 trauma autopsy reports, only 2.8% presented with tracheobronchial rupture, yet more than 80% of these patients died before reaching the hospital (1). The location of most traumatic tracheobronchial injuries occurred close to the carina, with approximately 58% of ruptures occurring within 1 cm of the carina and 76% within 2 cm of the carina, according to the review by Kiser and colleagues. Compared with tracheal and left bronchial injuries, right-sided bronchial injuries occurred most frequently, accounting for 47% of the 259 patients reported in the review; however, the incidence of middle lobe bronchial rupture is relatively rare, only about 1% (2). To our knowledge, incomplete laceration of the right middle lobe bronchus after blunt chest trauma as seen in our case has hardly been reported.

The clinical presentation of tracheobronchial rupture depends on whether there is free communication between the rupture site and the pleural cavity or mediastinum. The bronchial rupture in the chest cavity can lead to tension pneumothorax, subcutaneous emphysema as well as massive air leakage from the thoracostomy tube. However, if the ruptured bronchus does not communicate with the pleural cavity, the pneumothorax is not obvious and the main manifestation may be mediastinal emphysema (3, 4). In our case, the diagnosis of bronchial injury was really difficult because the patient arrived in an emergency with multiple injuries. He was in a state of shock with persistent hemopneumothorax because the fractured rib had punctured his right lung. At the time of emergency surgical exploration, we did not see any air leaks in the lung. Postoperatively, the lung was well recruited, and no air leak was noted from the right chest tube drainage. The injured bronchus relies solely on the surrounding sleeve tissue to maintain its function and the patient does not exhibit specific signs or symptoms.

A full understanding of the mechanisms and conditions under which tracheal and bronchial injuries occur is required to raise a high level of suspicion for the diagnosis of bronchial rupture (5). From the studies of Kirsh (1), we have learned about the possible mechanisms of tracheobronchial injury. (1) Sudden compression of the chest by an external force shortens the anterior and posterior thoracic diameters and lengthens the transverse diameter. Lateral pulling tears the bronchi, which mostly occurs in the tracheal carina. (2) Sudden increase in airway pressure, especially when the epiglottis is closed. The pressure in the trachea and bronchi increases significantly, resulting in airway membrane laceration. (3) Rapid deceleration in motor vehicle accidents or fall injuries, where the airway is fixed in the neck and mediastinum while the lung is easily moved in the pleural cavity, and this mechanism produces a shear force that causes tracheal and bronchial rupture. Airway injuries such as the one in our case present with nonspecific signs and symptoms that can be very misleading if not combined with the medical history and the mechanisms described above.

The diagnosis of incomplete tracheobronchial injury is difficult and is usually delayed by the lack of specific presentations. In patients with suspected incomplete bronchial rupture, chest CT with 3D reconstruction can be helpful since it can directly show bronchial rupture. Atelectatic lung tissue, however, may interfere with the judgment. In our current case, through the initial CT scan, the laceration was not observed due to the collapse of the right lungs. It was not until the CT was repeated 4 days after surgery that a minor discontinuity of the right middle lobe endobronchial wall was found in the axial and sagittal view (Figures 1A,B), which arose our great attention and suspicion of a bronchus rupture. Meanwhile, a bedside bronchoscopy revealed an incomplete laceration of the right middle lobe bronchus after removing secretions and sputum crust from the patient's airway (Figure 1D). Bronchoscopy can be used as the gold standard for diagnosing and describing airway injury. It can effectively assess the topography, extent, and depth of the lesion and evaluate the airway and digestive tract. However, sometimes fissures may be difficult to visualize due to the presence of inflammation and airway secretions. Bronchoscopy can also be performed in the operating room for better patient cooperation, and more importantly, it can help with difficult intubation or stent placement at the lesion.

Treatment of patients with bronchial laceration varies from person to person. Once diagnosed, those who meet the criteria should have early surgery, and if possible, the sooner the laceration is repaired, the better. Surgical treatment of patients with bronchial laceration can rapidly restore bronchial continuity, terminate pneumothorax and mediastinal emphysema, and reduce the incidence of complications caused by bronchial ruptures, such as pulmonary infection, pulmonary solidus, and tracheobronchial stenosis (2). In addition, bronchoscopy should be used for intensive observation and follow-up. It is necessary to aspirate the secretions accumulated around the bronchi with repeated bronchoscopy to accelerate recovery and obtain more information. After multidisciplinary consultation we decided to remove the patient's right middle lobe for the following reasons: our case was diagnosed late and the infection was severe; the incomplete bronchial rupture was along the long axis of the trachea and was relatively wide and deep, making it difficult to repair; and the repair was likely to cause bronchial stenosis in the distant future leading to conditions such as atelectasis; in addition, the patient required surgery on the spine and intraoperative tracheal intubation might aggravate the bronchial rupture.

## Conclusion

In conclusion, tracheobronchial injuries with incomplete lacerations after trauma may be overlooked because of the lack of specific clinical manifestations. Clinicians should give due consideration to this condition in the context of the patient's history and remain on high alert to avoid missed and delayed diagnoses. Three-dimensional reconstructed CT and fiberoptic bronchoscopy can be helpful in the diagnosis and treatment of these patients.

## Data Availability

The original contributions presented in the study are included in the article/Supplementary Material, further inquiries can be directed to the corresponding author/s.
